# Addenbrooke's Cognitive Examination-Revised is accurate for detecting dementia in Parkinson's disease patients with low educational level

**DOI:** 10.1590/S1980-57642014DN81000004

**Published:** 2014

**Authors:** Maria Sheila Guimarães Rocha, Elida Maria Bassetti, Maira Okada Oliveira, Roberta Gomes Borges Kuark, Nathercia Marinho Estevam, Sonia Maria Dozzi Brucki

**Affiliations:** 1 MD, PhD; Hospital Santa Marcelina, São Paulo, Brazil; 2 MD; Hospital Santa Marcelina, São Paulo, Brazil; 3 MsC. Hospital Santa Marcelina, São Paulo, Brazil

**Keywords:** Parkinson's disease, dementia, screening tests, neuropsychology, cognitive evaluation

## Abstract

Diagnosis of Parkinson's disease dementia is a challenge in clinical settings. A comprehensive neuropsychological evaluation is time-consuming and expensive; brief instruments for cognitive evaluation must be easier to administer and provide a reliable classification.

**Objective:**

To study the validity of the Brazilian version of Addenbrooke's Cognitive Examination-Revised (ACE-R) for the cognitive assessment of Parkinson's disease (PD) patients with heterogeneous educational level.

**Methods:**

Patients were evaluated according to the diagnostic procedures recommended by the Movement Disorder Society (MDS) as the gold standard for the diagnosis of dementia in PD.

**Results:**

We studied 70 idiopathic PD patients, with a mean (SD) age of 64.1 (9.3) years and mean disease duration of 7.7 (5.3) years and educational level of 5.9 years, matched for education and age to controls. Twenty-seven patients fulfilled MDS clinical criteria for PD dementia. Mean scores on the ACE-R were 54.7 (12.8) points for patients with PD dementia, 76 (9.9) for PD patients without dementia and 79.7 (1.8) points for healthy controls. The area under the receiver operating curve, taking the MDS diagnostic procedures as a reference, was 0.93 [95% CI, 0.87-0.98; p<0.001] for ACE-R. The optimal cut-off value for ACE-R was ≤72 points [sensitivity 90%; specificity 85%; Kappa concordance (K) 0.79].

**Conclusion:**

ACE-R appears to be a valid tool for dementia evaluation in PD patients with heterogeneous educational level, displaying good correlation with clinical criteria and diagnostic procedures of the MDS.

## Abstract (pt)

O diagnóstico da demência na doença de Pakinson é desafiador na prática clínica. A avaliação neuropsicológica ampla é cara e demorada; instrumentos breves para avaliação cognitiva devem ser fáceis de realizar e fornecer uma classificação confiável.

**Objetivo:**

Estudar a validade da versão Brasileira do *Addenbrooke's Cognitive Examination-Revised* (ACE-R) para a avaliação em pacientes com doença de Parkinson (DP) com nível de escolaridade heterogêneo.

**Métodos:**

Os pacientes foram avaliados segundo os procedimentos diagnósticos recomendados pela *Movement Disorder Society* (MDS) como padrão ouro para diagnóstico de demência da DP (DDP).

**Resultados:**

Nós estudamos 70 pacientes com DP idiopática, pareados por idade e escolaridade a controles, com média de idade de 64,1 (9,3) anos, tempo médio de doença de 7,7 (5,3) anos e nível educacional de 5,9 anos. Vinte e sete pacientes preencheram critério da MDS para DDP. Os escores médios na ACE-R foram de 54,7 (12,8) pontos para DDP, 76 (9,9) para DP sem demência e 79,7 (1,8) pontos para os controles saudáveis. A área sob a curva tomando-se os procedimentos diagnósticos da MDS como referência foi 0,93 [95% CI, 0,87-0,98; p<0,001] para ACE-R. O melhor escore de corte foi de ≤72 pontos (sensibilidade de 90% e especificidade de 85%; Kappa concordance (K) 0.79).

**Conclusão:**

A ACE-R parece ser um instrumento válido para avaliação de demência em pacientes com DP de níveis educacionais heterogêneos, mostrando boa correlação com o critério clínico e procedimentos diagnósticos da MDS.

## INTRODUCTION

Parkinson's disease (PD) is a chronic degenerative disease characterized by motor symptoms, such as tremor, rigidity and bradykinesia,^1^ and non-motor symptoms, including sensory and neuropsychiatric disorders.^2^ Emotional and cognitive disorders associated with PD are increasingly recognized as equally, or more, debilitating than motor symptoms. PD patients have a six-fold increased risk of developing dementia compared with healthy controls, and 3-4% of dementia cases in the general population are due to PD.^3^ The point prevalence of dementia associated with Parkinson's disease (PDD) has been estimated to range from 20% to 40%.^4-6^ This wide variability is due to several factors, including the assessment method used, with a higher prevalence reported in studies using comprehensive neuropsychological instruments compared with those screening global cognitive function,^7^ and the proportion of patients with multiple risk factors for dementia development.^8^

According to a consensus published by the Movement Disorder Society Task Force in 2007,^9^ the diagnosis of dementia in PD should rely firstly on the PD fulfilling the UK PD Society Brain Bank criteria.^10^ Secondly, the disease should have developed prior to the onset of dementia, and patients must present decreased global cognitive efficiency, with more than one cognitive domain (memory, attention, visuo-constructional ability and executive function) affected, and impairment of daily life activities. Risk factors for the development of PDD include: increasing age, older age at onset of disease, longer disease duration, severity of parkinsonism, male gender and presence of psychiatric symptoms.^11^

A full cognitive ability evaluation in PD is time consuming, might be exhausting for the patient, and is unfortunately not fully available in most low-income and developing countries. The need for brief, sensitive and specific cognitive screening instruments clearly exists. The Mini-Mental State Examination (MMSE) has been proposed as a first-line assessment tool for global cognitive efficiency in PD because of its simplicity and wide use in dementia.^9^ However, early cognitive deficits in PD, such as executive dysfunction, frequently go undetected by the MMSE, limiting its usefulness.^12^ The Scales for Outcomes of Parkinson's disease-Cognition (SCOPA-COG) were developed with the purpose of serving as a short and practical instrument, but are sensitive only to specific cognitive deficits in PD. This tool has undergone only partial validation, thus further reducing its applicability.^13,14^ Finally, Addenbrooke's Cognitive Examination-Revised (ACE-R) is a brief yet reliable test battery that provides evaluation of six cognitive domains (orientation, attention, memory, verbal fluency, language and visuospatial ability).^15^ It was developed and revised to provide a brief test sensitive to the early stages of dementia, and is also effective for differentiating the subtypes of dementia, such as Alzheimer's disease, frontotemporal dementia, progressive supranuclear palsy, and other forms of dementia associated with parkinsonism.^16-19^ The first version of ACE has been validated for PD patients^20^ and ACE-R has recently been used in two other PD studies,^21,22^ revealing good discriminative properties compared with neuropsychological evaluation as a reference for the diagnosis of dementia. None of the studies used the diagnostic procedures for PD dementia recommended by the Movement Disorder Society (MDS) nor have samples with low educational level. For this reason, we established this protocol to study the validity of ACE-R in the initial assessment of global cognitive efficiency in PD, taking the diagnostic procedures for PDD recommended by the MDS Task Force^9^ as a reference method.

## METHODS

**Study sample.** In this study, 70 consecutive PD outpatients from our tertiary movement disorders clinic at Santa Marcelina Hospital were assessed. Only idiopathic PD patients were included, using the UK PD Society Brain Bank criteria.^10^ Following the recommendations of the MDS Task Force for the diagnosis of dementia, patients who presented with major depression, delirium or other abnormalities that could obscure the diagnosis of dementia were excluded.^9^ Cases of major depression were also excluded, as defined by the Diagnostic and Statistical Manual of Mental Disorders-IV (DSM-IV) criteria, and by scores higher than 18 on the Beck Depression Inventory (BDI).^23^ Other exclusion criteria were: cognitive decline secondary to systemic, vascular or other degenerative disease; history of drug and alcohol abuse; previous neurosurgical procedure or traumatic brain injury; and use of drugs with anticholinergic effects. These same exclusion criteria (depression, cognitive impairment, drug abuse, TBI, and neurosurgery) were applied to the healthy controls, matched to the PD patients for age and educational level, having MMSE scores higher than median scores for educational level.^24^ The healthy control group comprised caregivers, family members and members from community invited to participate in this study, all of whom granted informed consent.

**Patient evaluation.** Patients were initially evaluated using the Unified Parkinson's Disease Rating Scale (UPDRS),^25^ the Hoehn and Yahr scale (H&Y),^26^ and the Schwab & England daily activity scale (S&E).^27^ A structured clinical interview was applied to record demographic data and take a medical and drug history. The PD clinical subtypes were then classified into: tremor dominant and PIGD (postural instability gait disorder) dominant type, according to scores on the UPDRS sub-items.^28^

The neuropsychological evaluation comprised tests recommended by the MDS (Level II) and tests validated for the Brazilian population. The battery included: the MMSE;^24^ visual reproduction and logical memory subtests of the Wechsler Memory Scale Revised (WMS-R);^29^ the Rey Auditory Verbal Learning Test (RAVLT);^30^ the Block Design subtest of the Wechsler Adult Intelligence Scale (WAIS),^29,31^ the Rey-Osterrieth Complex Figure test (copy and delayed recall);^32^ the Trail Making Test parts A and B; the Stroop Test; verbal fluency (both phonemic, 'F-A-S', and categorical, e.g. animals); and the Frontal Assessment Battery.^33,34^ In order to avoid fatigue in the PD patients, the neuropsychological assessment was conducted over two visits, each lasting approximately 90 minutes. The neuropsychologist who performed the neuropsychological evaluation was blinded to the ACE-R results, and independent neurologists applied the ACE-R scale and MDS clinical criteria for PD dementia^9^ after analysis of NPS data. Taking account of the MDS clinical criteria for dementia in PD, the patients were classified into two groups: those with dementia (PDD) and those without dementia (PDwD).

ACE-R evaluates six cognitive domains totaling 100 points: orientation (10 points), attention (8 points), memory (35 points), verbal fluency (14 points), language (28 points) and visuospatial abilities (5 points). The maximum possible score is 100. The adapted Brazilian version of ACE-R was used in the present study.^35^ The Research Ethics Committee of the Hospital Santa Marcelina approved the protocol, and all participants signed a free informed consent form prior to study entry.

**Statistical analysis.** Scale scores were correlated by nonparametric Spearman's rho coefficient. Receiver operating curve (ROC) analysis was employed for ACE-R and for MMSE diagnostic performance evaluation, with MDS clinical criteria used as the reference method. Finally, sensitivity, specificity and kappa concordance values (K) were calculated for several cut-off values. The cut-off points with the highest sensitivity, specificity and K values were selected as the optimum cut-off point values for dementia diagnosis. Mean scores were compared using Student's t-test, and Fisher's exact test was employed to perform frequency comparisons. Linear regression analysis was used to quantify variable correlations. Alpha was set at 0.05. Both data management and statistical analysis were carried out using GraphPad^©^ Prism 5 software (GraphPad Software, Inc., CA, USA).

## RESULTS

A total of 70 PD patients were evaluated, predominantly males and with educational level ranging from 2 to 16 years of schooling. Table 1 compares demographic data, clinical characterization, and scores on the MMSE and ACE-R.

**Table 1:** Demographic and clinical data in PD subjects without and with dementia.

		PD patients	Without dementia n=43	With dementia n=27	p values*
Males (%)		40 (57.1)	28 (65.1)	11 (40.7)	0.039
Age (years)		64.1 (9.3)	61.83	67.48	0.012
Age at onset (years)		56.4 (10.1)	54.9	58.7	0.131
Education (years)		5.9 (3.4)	7.3	3.9	< 0.001
Disease duration (years)		7.7 (5.3)	7.16	8.9	0.189
UPDRS I		10.5 (8.1)	6.8	14.2	< 0.001
UPDRS II		15.7 (8.2)	12.6	22.2	<0.001
UPDRS III		39.4 (18.9)	34.1	48.1	0.003
UPDRS IV		4.5 (4.8)	2.6	5.8	0.040
UPDRS total		67.7 (32.3)	56.3	90.1	<0.001
Hoehn & Yahr (mean)		2.4	2.1	3.1	0.001
	H&Y I-II (N)	38	38	5	<0.001
	H&Y III-V (N)	32	5	22	<0.001
Schwab & England (5)		77.5	83.6	67.7	<0.001
L-dopa therapy (years)		5.8 (4.9)	5.28	6.3	0.395
PD clinical subtype	Tremor dominant (%)	46 (65.7%)	31 (72.2)	15 (55.6)	0.123
	PIGD dominant (%)	14 (20%)	6 (13.9)	8 (29.6)	0.099
	Undetermined (%)	10 (14.3%)	6 (13.9)	4 (14.8)	0.134
MMSE score		24.4 (4.2)	26.7 (1.9)	20.7 (3.9)	<0.001
ACE-R score (SD)		67.8 (15.3)	76 (9.9)	54.7 (12.8)	<0.001
ACE-R subdomain (SD)	Attention - Orientation	15.1 (3.1)	16.6 (1.5)	12.5 (2.7)	<0.001
	Memory	13.8 (4.9)	15.9 (4.3)	10.8 (4.2)	<0.001
	Verbal fluency	7.1 (2.5)	8.1 (2.1)	5.4 (2.3)	<0.001
	Language ability	21.6 (5.4)	23.5 (3.3)	17.1 (6.1)	<0.001
	Visuospatial ability	11.3 (3.9)	12.4 (2.7)	8.7 (4.4)	<0.001

ACE-R: Addenbrooke's Cognitive Examination-Revised; MMSE: Mini-Mental State Examination; PIGD: postural instability gait disorder; UPDRS: Unified Parkinson's Disease Rating Scale, part I mentation, part II daily activities, part III motor evaluations, part IV levodopa complications.

* p-values from comparisons of PD w/o dementia and PD w/ dementia.

There was no difference between PD patients and controls in terms of age and education, with respective means in controls of 62.3 (8.9) years of age and 6.9 (4.2) years of schooling. However, there was a significant difference between PD patients and controls on MMSE scores and ACE-R scores (for total and on all sub-items. The scores by controls were ACE-R total score - 79.7 (7.5); attention-orientation - 16.8 (1.7); memory - 16.6 (4.3); verbal fluency - 8.9 (2.7); language skill - 23.7 (2.6); and visuospatial skill - 13.6 (1.9).

Twenty-seven PD patients (38.6%) were diagnosed as PDD using the MDS criteria. Cognitive dysfunction was significantly more frequent at worse severity stages (H&Y 3-4): 22 out of 27 patients (Yates corrected Chi square=31.27; p<0.001).

ACE-R total score was negatively correlated with H&Y stage (r= -0.53; p=0.011) and linear regression analysis confirmed the impact of H&Y stage on ACE-R total score, with a reduction of 8.74 points for each increasing stage on the H&Y scale (coefficient= -8.744; SE=1.94; 95% CI: -12.6 to -4.85; F-test=20.16 with 68 df; p<0.0001). Further correlation analysis revealed a mild positive correlation with schooling (r=0.47; p=0.041) and S&E scale score (r=0.49; p=0.032), and a mild negative correlation with the UPDRS total score (r=0.47; p=0.037). Amongst 62 healthy controls aged 47-82 years, observed a positive correlation was observed between ACE-R total score and educational level (r=0.61; p=0.001).

**Figure 1:**
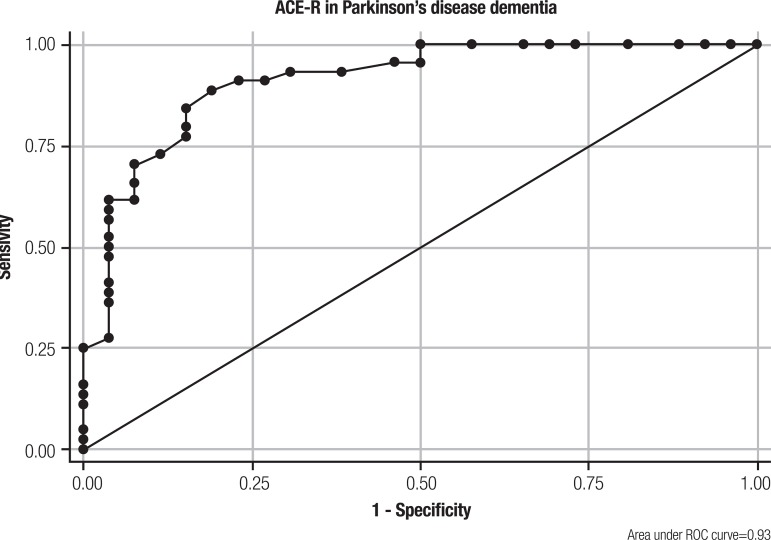
Receiver operating curve relating to the diagnostic performance of Addenbrooke's Cognitive Examination-Revised (ACE-R) for dementia in PD.

Positive and significant correlations were detected between ACE-R and MMSE scales (r=0.84; p<0.001). Using the MDS diagnostic procedures for dementia in PD as the reference method, for ACE-R, the area under the ROC curve was 0.93 (95% CI: 0.86-0.98; p<0.0001; Figure 1), and for MMSE, the area was 0.88 (95% CI: 0.78-0.97; p<0.001). For ACE-R, the optimum cut-off value was ≤72 points [sensitivity=89.3% (95% CI: 71.8-97.7%); specificity=84.6% (95% CI: 71.9-93.1%); K=0.79], and for MMSE, the value was 24 points [sensitivity=78.5% (95% CI: 59.1-91.7%); specificity=96.4% (95% CI: 81.6-99.9%); K=0.69].

## DISCUSSION

As therapeutic approaches for PD dementia are now available, there is clearly a need for brief, sensitive and specific cognitive screening instruments. The Movement Disorder Society Task Force on PD dementia^9^ has proposed that, for those cases in which dementia diagnosis remains uncertain or equivocal after the first level of evaluation, a second level should be executed using more specific cognitive tests in order to specify the pattern and severity of the dementia. Second-level evaluations consist of a series of qualitative tests that allow for a more comprehensive assessment of cognitive functions, such as the MMSE. Comparison between diagnostic criteria (Level II) and clinical procedures (Level I) for PD dementia has revealed that Level II had good discrimination in the detection of PD dementia, whereas Level I criteria had lower sensitivity (31.25%), greater specificity (90.19%), and positive and negative predictive values of 50% and 82.45%, in the detection of PD dementia.^6^ The lower sensitivity with Level I criteria could be related to the adoption of an MMSE cut-off value of less than 26. This suggests that the MMSE cut-off value proposed by MDS Level I criteria could be affected by educational level, and not considering educational level could lead to a false-negative PD dementia diagnosis. Although the MMSE has been recommended as a useful tool for identifying cognitively impaired PD patients, some studies have called into question its accuracy for detecting cognitive impairments in PD.^12^

A previous study using the first version of ACE in PD patients showed that the test had excellent correlation with both comprehensive and validated tools, such as the Mattis Dementia Rating Scale, as well as with the PD-specific scale SCOPA-COG, which ultimately proved superior to the MMSE regarding its clinometric properties in PD patients. In the former study, the ACE cut-off scores were set at 83 points, revealing sensitivity and specificity in PD patients of 92% and 90%, respectively. A second study considered two cut-off points according to patient age: a similar optimal cut-off value of 83 points for the ACE-R for the young group, which is in accordance with the index article,^15^ and an optimal cut-off of 75 points for the old group, which is in agreement with the results of a recent study by Larner et al. examining optimal cut-off values for ACE-R in everyday clinical practice.^36^ In this present study, we have an optimal cut-off point of 72 points when the entire group of PD patients is considered. In both of the previously cited studies, and also in the index article, the patients' educational level was much higher than that observed in our sample, which might have influenced the ACE-R scores in this series.^15^ The observed correlation indices between ACE-R and years of schooling reinforce this argument, not only in the group of PD patients but also in the healthy controls. Further studies concerning the impact of educational level on the ACE-R score are required to confirm these findings.

Attention, working memory, visuospatial and executive functions are especially impaired in PD, whereas verbal functions, thinking and reasoning are relatively spared.^3^ Both the MMSE and ACE-R evaluate many of these functions; however, the differences between them are striking. In line with this, memory evaluation forms only a small part of the MMSE - accounting for only 10% of the total score - whereas one-third of ACE-R relates to memory. In addition, ACE-R allows a better evaluation of verbal fluency, serial learning, and extended language by adding 10 objects to the naming task, and assigning greater depth to reading evaluation, as well as including a more stringent comprehension test. The clock and cube drawings added to the MMSE pentagon-drawing task have enriched its visuoconstructional function evaluation.^15^ A comprehensive neuropsychological evaluation, according to the MDS, takes about two hours to complete. ACE-R has the advantage of being less time-consuming (approx. 20 min), and produced an area under the curve of 0.93, with good accuracy for differentiating PD patients with dementia from those without.

In conclusion, our results suggest that, taking the gold standard as a reference, a well-established and comprehensive cognitive battery for dementia diagnosis in PD,^9^ ACE-R has proved to be an appropriate instrument, with very good sensitivity and specificity, for first-line global evaluation of cognitive deficits in PD patients.
